# A Novel Scoring System for Prediction of Disease Severity in COVID-19

**DOI:** 10.3389/fcimb.2020.00318

**Published:** 2020-06-05

**Authors:** Chi Zhang, Ling Qin, Kang Li, Qi Wang, Yan Zhao, Bin Xu, Lianchun Liang, Yanchao Dai, Yingmei Feng, Jianping Sun, Xuemei Li, Zhongjie Hu, Haiping Xiang, Tao Dong, Ronghua Jin, Yonghong Zhang

**Affiliations:** ^1^Beijing You'an Hospital, Capital Medical University, Beijing, China; ^2^Chinese Academy of Medical Science Oxford Institute (COI), University of Oxford, Oxford, United Kingdom; ^3^MRC Human Immunology Unit, MRC Weatherall Institute of Molecular Medicine, University of Oxford, Oxford, United Kingdom

**Keywords:** logistic regression, severity pneumonia, COVID-19, retrospective cohort, prediction scoring system

## Abstract

**Background:** A novel enveloped RNA beta coronavirus, Corona Virus Disease 2019 (COVID-19) caused severe and even fetal pneumonia in China and other countries from December 2019. Early detection of severe patients with COVID-19 is of great significance to shorten the disease course and reduce mortality.

**Methods:** We assembled a retrospective cohort of 80 patients (including 56 mild and 24 severe) with COVID-19 infection treated at Beijing You'an Hospital. We used univariable and multivariable logistic regression analyses to select the risk factors of severe and even fetal pneumonia and build scoring system for prediction, which was validated later on in a group of 22 COVID-19 patients.

**Results:** Age, white blood cell count, neutrophil, glomerular filtration rate, and myoglobin were selected by multivariate analysis as candidates of scoring system for prediction of disease severity in COVID-19. The scoring system was applied to calculate the predictive value and found that the percentage of ICU admission (20%, 6/30) and ventilation (16.7%, 5/30) in patients with high risk was much higher than those (2%, 1/50; 2%, 1/50) in patients with low risk (*p* = 0.009; *p* = 0.026). The AUC of scoring system was 0.906, sensitivity of prediction is 70.8%, and the specificity is 89.3%. According to scoring system, the probability of patients in high risk group developing severe disease was 20.24 times than that in low risk group.

**Conclusions:** The possibility of severity in COVID-19 infection predicted by scoring system could help patients to receiving different therapy strategies at a very early stage.

**Topic:** COVID-19, severe and fetal pneumonia, logistic regression, scoring system, prediction.

## Introduction

A cluster of cases of acute respiratory illness with unknown etiology was reported in Wuhan City, Hubei Province of China from December 2019 (Chen et al., 2020). The pathogen was identified as a novel enveloped RNA beta coronavirus by the Chinese Center for Disease Control and Prevention (CDC) (Wu et al., 2020), and was designated as severe acute respiratory syndrome coronavirus 2 (SARS-CoV-2) (Zhu et al., 2020). The World Health Organization (WHO) declared the novel coronavirus disease, COVID-19; a public health emergency of international concern, and by 11 March 2020, the COVID-19 outbreak was declared a global pandemic. According to Coronavirus disease 2019 (COVID-19) situation report from WHO, totally 191,127 cases of patients were laboratory confirmed and amongst them 7,807 patients died by 18th March 2020 (Liu T. et al., 2020; World Health Organization, 2020).

Infection in the majority of people is mild, with common clinical characteristics including fever, cough, and sputum. Some infected patients also reported gastrointestinal symptoms including vomiting and diarrhea (Perlman and Netland, 2009; Fehr and Perlman, 2015). Dyspnea and/or hypoxemia occurred after 1 week, with 50% of severe patients quickly progressing to acute respiratory distress syndrome, septic shock, refractory metabolic acidosis, coagulation disorders, and multi-organ failure, even life-threatening (China National Health Commission, 2020). However, there is still no clear critical predictive factors and models to prognosticate the severity of the disease. This article intends to conduct a group study of 80 patients with COVID-19 infection in a tertiary teaching hospital specializing on infectious diseases to screen for critical factors related to the disease and establish a predictive model for disease severity. Early detection of severe patients with COVID-19 is of great significance to shorten the disease course and reduce mortality.

## Materials and Methods

### Study Population

Patients were recruited from Beijing You'an Hospital, Capital Medical University, Beijing. A discovery cohort (80 cases) was setup between January 2020 and February 2020 and a validation group (22 cases) was setup from March to April of 2020. All participants were hospitalized patients with laboratory-confirmed COVID-19. Their clinical data was collected from Electronic Medical Record System (EMRS), Laboratory Information System (LIS) and Picture Archiving and Communication System (PACS). The study was approved by the Institutional Review Board of Beijing You'an Hospital.

### Clinical Definitions

COVID-19 was diagnosed according to the diagnosis and treatment of coronavirus disease 2019 (COVID-19) recommended by the National Health Commission of China (China National Health Commission, 2020). The laboratory-confirmed patient was defined as a positive result on high throughput sequencing or real-time reverse-transcriptase-polymerase-chain-reaction (RT-PCR) assay of nasal and pharyngeal swab specimens. The degree of severity was divided as mild infection and severe infection. Severe infection was defined as COVID-19 confirmed patients with one of conditions: respiratory distress with RR>30/min; Blood oxygen saturation <93%; arterial oxygen partial pressure (PaO_2_)/Fraction of inspired O_2_ (FiO_2_) <300 mmHg; respiratory failure with mechanical ventilation; shock; or other organ failures need intensive care in ICU. Initial stage of COVID-19 infection was defined as patients during their first week of infection only with the common clinical characteristics, such as fever, cough, sputum, vomit, and diarrhea.

### Treatment Procedure and End-Point of Observation

All of patients received standard therapy according to the “Diagnosis and Treatment of Coronavirus Disease 2019” guidelines recommended by the National Health Commission of China (China National Health Commission, 2020). The observed end-point was defined as recovery or death in 28 days in hospital.

### Clinical Observed Variables

A total of 48 indicators were collected from the candidates at the initial stage of COVID-19 infection, including age, gender, pre-existing conditions (respiratory disease, cardiac disease, hypertension, hyperlipemia, diabetes, kidney disease, liver disease, post-operative, and more than two kinds of diseases), presenting symptoms (fever, cough, expectoration, vomit, and diarrhea). Laboratory detections at the initial stage of COVID-19 infection included pH, partial pressure of carbon dioxide (PCO_2_), partial pressure of oxygen (PO_2_), blood oxygen saturation (SaO_2_), white blood cell count (WBC), hemoglobin (HGB), platelet count (PLT), absolute value of lymphocyte (LYM), absolute value of monocyte (MONO), absolute value of neutrophil (NEU), lymphocyte percentage (LYM%), neutrophil percentage (NEU%), ratio of neutrophil to lymphocyte (NLR), prothrombin time (PT), prothrombin activity (PTA), fibrinogen content (FIB), procalcitonin (PCT), c-reactive protein (CRP), alanine aminotransferase (ALT), aspartate aminotransferase (AST), total bilirubin (TBIL), albumin (ALB), creatinine (Cr), glomerular filtration rate (GFR), carbon dioxide combining power (CO2CP), creatine kinase (CK), creatine kinase isoenzyme-MB (CK-MB), myoglobin, troponin, and lactic acid. Computerized Tomography (CT) imaging was employed to evaluate the ground-glass opacity (GGO).

### Statistical Analysis

Statistical analysis of the categorical data was performed using the Chi-square test. Fisher's exact test was used since the Chi-square approximation might not hold for the relatively small sample size. Student's *t*-test was used to compare continuous values between mild and severe infection groups in which case data were normally distributed (evaluated with Kolmogorov-Smirnov test), and non-parametric *t*-test (Mann-Whitney test) was used when data were not normally distributed. The univariate and multivariate logistic regression analysis of variables potentially associated with severity of COVID-19 infection. The optimal cutoff values were calculated in accordance with the receiver operating characteristic curves and Youden's index. The prediction value of scoring system was determined by the area under the curve (AUC). Statistical test differences were considered significant if the *P*-values were <0.05. Analyses were performed with SPSS software v 25.5 (IBM, NY, USA).

## Results

### Clinical and Laboratory Characteristics of Discovery Cohort

Eighty hospitalized patients with laboratory-confirmed COVID-19 were recruited in the study in total, and all candidates were divided into those with “mild” and “severe” disease according to the clinical definitions from the National Health Commission of China. Mild disease (*n* = 56) was defined as those with fever, respiratory symptoms and pneumonia from imaging. Patients with severe disease (*n* = 24) were those with the symptoms described above, but deteriorated and developed respiratory distress or respiratory failure. Blood oxygen saturation in the patients (24/24) in the severe group was below 93%, and none of 56 patients in mild group was below 93%. The ratio of arterial oxygen partial pressure (PaO_2_) to Fraction of inspired O_2_ (FiO_2_) was 223.5 ± 45.77 mmHg in severe group, much lower than that in mild group (466.7 ± 135.6 mmHg, *p* < 0.001). Seven patients in severe group received intensive care in ICU, 6 patients mechanically ventilated, and among them three severely infected patients died. Demographic data are shown in Table 1.

**Table 1:** Patient demographics and clinical phenotype.

	**All hospitalized patients (*n =* 80)**	**Mild disease (*n =* 56)**	**Severe (*n =* 24)**	**Died (*n =* 3)**	***P*-value^**a**^**
Age, mean ± SD, yr	51.16 ± 17.476	45.34 ± 15.25	64.75 ± 14.76	84.00 ± 8.185	1.0E-06
Gender, Men, *n*/total (%)	33 (41.25%)	24 (42.86%)	9 (37.5%)	1 (33.3%)	0.656
**Pre-existing conditions**, ***n*****/total (%)**					
Respiratory diseases	4/80 (5%)	0/56 (0%)	4/20 (16.7%)	1/3 (33.3%)	6.7E-03
Cardiac diseases	9/80 (11.25%)	3/56 (5.4%)	6/24 (25%)	3/3 (100%)	0.0186
Hypertension	18/80 (22.5%)	7/56 (12.5%)	11/24 (45.8%)	3/3 (100%)	1.07E-03
Hyperlipemia	3/80 (3.8%)	2/56 (3.6%)	1/24 (4.2%)	1/3 (33.3%)	1.000
Diabetes	9/80 (11.25%)	5/56 (8.93%)	4/24 (16.67%)	1/3 (33.33%)	0.441
Kidney diseases	2/80 (2.5%)	0/56 (0%)	2/24 (8.3%)	1/3 (33.3%)	0.087
Liver diseases	5/80 (6.2%)	2/56 (3.6%)	3/24 (12.5%)	0/3 (0%)	0.156
Post-operative	16/80 (20%)	12/56 (21.4%)	4/24 (16.7%)	1/3 (33.3%)	0.765
Other diseases	6/80 (7.5%)	3/56 (5.4%)	3/24 (12.5%)	0/3 (0%)	0.358
More than 2 kinds of diseases	19/80 (23.8%)	8/56 (14.3%)	11/24 (45.8%)	0/3 (0%)	2.38E-03
**Presenting symptoms**, ***n*****/total (%)**					
Fever	62/80 (77.50%)	41/56 (73.21%)	21/24 (87.50%)	2/3 (66.67%)	0.161
Cough	51/80 (63.75%)	33/56 (58.93%)	18/24 (75.00%)	2/3 (66.67%)	0.171
Expectoration	26/80 (32.50%)	15/56 (26.79%)	11/24 (45.83%)	2/3 (66.67%)	0.096
Vomit	1/80 (1.25%)	0/56 (0%)	1/24 (4.17%)	0/3 (0%)	0.300
Diarrhea	1/80 (1.25%)	1/56 (1.79%)	0/24 (0%)	0/3 (0%)	1.000
**1st laboratory detection, mean** **±** **SD**					
PH	7.42 ± 0.06	7.42 ± 0.05	7.41 ± 0.07	7.32 ± 0.14	0.471
PCO2	33.79 ± 6.69	34.43 ± 7.23	32.26 ± 4.99	30.37 ± 4.80	0.264
PO2	96.44 ± 31.37	95.86 ± 32.99	97.83 ± 27.96	104.37 ± 20.59	0.830
SaO2	94.65 ± 5.69	94.74 ± 4.49	94.42 ± 8.08	91.93 ± 10.71	0.847
WBC	4.73 ± 1.81	4.15 ± 1.37	6.08 ± 2.02	4.94 ± 2.81	1.5E-04
HGB	131.97 ± 22.20	131.51 ± 24.13	133.04 ± 17.31	133.67 ± 11.02	0.780
PLT	215.34 ± 97.63	211.52 ± 81.87	224.25 ± 128.81	204.00 ± 121.87	0.596
LYM	1.22 ± 1.07	1.33 ± 1.22	0.95 ± 0.50	0.48 ± 0.29	0.152
MONO	0.37 ± 0.42	0.36 ± 0.49	0.36 ± 0.17	0.36 ± 0.23	0.994
NEU	3.17 ± 2.04	2.40 ± 1.25	4.96 ± 2.41	4.12 ± 2.04	3.3E-05
LYM%	26.07 ± 12.51	30.09 ± 11.51	16.68 ± 9.41	9.53 ± 3.00	3.0E-06
NEU%	63.73 ± 14.23	59.03 ± 12.43	74.67 ± 12.16	68.70 ± 20.66	2.0E-06
NLR	4.08 ± 5.33	2.56 ± 1.99	7.63 ± 8.32	7.85 ± 4.14	6.9E-03
PT	12.69 ± 1.10	12.69 ± 1.01	12.72 ± 1.32	12.10 ± 2.16	0.914
PTA	75.56 ± 9.46	75.32 ± 7.91	76.13 ± 12.66	84.67 ± 26.65	0.732
FIB	3.43 ± 1.04	3.27 ± 1.03	3.80 ± 0.98	3.47 ± 0.87	0.041
PCT	0.12 ± 0.06	0.12 ± 0.04	0.14 ± 0.09	0.27 ± 0.18	0.199
CRP	31.79 ± 40.79	20.30 ± 24.99	58.59 ± 56.15	87.30 ± 73.40	3.4E-03
ALT	38.32 ± 36.18	40.53 ± 41.19	33.17 ± 19.94	13.00 ± 6.08	0.408
AST	38.43 ± 30.59	37.67 ± 33.71	40.21 ± 22.22	35.00 ± 22.11	0.736
TBIL	10.85 ± 5.35	9.82 ± 4.19	13.27 ± 6.89	18.60 ± 7.35	7.4E-03
ALB	36.06 ± 4.86	37.48 ± 3.88	32.76 ± 5.37	33.60 ± 5.38	4.5E-04
Cr	72.61 ± 40.15	72.35 ± 42.69	73.21 ± 34.32	94.00 ± 23.43	0.931
GFR	97.74 ± 25.14	102.64 ± 24.16	86.28 ± 24.06	55.97 ± 17.18	6.8E-03
CO2CP	26.50 ± 3.26	26.53 ± 3.26	26.44 ± 3.32	23.07 ± 1.32	0.908
CK	135.26 ± 212.84	132.19 ± 236.75	142.42 ± 146.58	176.67 ± 153.88	0.845
CK-MB	0.67 ± 0.88	0.48 ± 0.65	1.09 ± 1.17	2.37 ± 0.82	2.3E-02
Myoglobin	70.13 ± 81.02	46.28 ± 33.53	125.75 ± 123.48	297.00 ± 201.31	4.8E-03
Troponin	0.02 ± 0.05	0.01 ± 0.01	0.05 ± 0.08	0.18 ± 0.20	3.0E-02
Lactic acid	1.37 ± 0.61	1.27 ± 0.49	1.57 ± 0.79	2.67 ± 1.16	0.104
**Imaging of CT scan**, ***n*****/total (%)**					
GGO	74/80 (92.5%)	51/56 (91.1%)	23/24 (95.8%)	3/3 (100%)	0.663
**DIAGNOSIS MARKERS OF SEVERITY**					
**Physiological variables, median (IQR)**					
RR	20 (20–21)	20 (20–20)	21 (20–24.75)	23 (20–25)	2.03E-04
SaO_2_(*n =* 56)	94.95 (88.125–97.625)	97.2 (95.5–98.1)	88.0 (79.6–90.9)	79.6 (77.6–80.3)	<1.0E-06
P/F(*n =* 45)	386.5 (261.85–472.0)	449.5 (379.1–494.3)	211.35 (192–260)	193.8 (187–200)	<1.0E-06
ICU admission, *n*/total (%)	7/80 (8.75%)	0/56 (0%)	7/24 (29.17%)	3/3 (100%)	1.09E-04
Mechanical ventilation, *n*/total (%)	6/80 (7.5%)	0/56 (0%)	6/24 (25.00%)	3/3 (100%)	4.48E-04
28 days mortality, *n*/total (%)	3/80 (3.75%)	0/56 (0%)	3/24 (12.5%)	3/3 (100%)	0.025

*PCO^2^, partial pressure of carbon dioxide; PO_2_, partial pressure of oxygen; SaO2, blood oxygen saturation; WBC, white blood cell count; HGB, hemoglobin; PLT, platelet count; LYM, absolute value of lymphocyte; MONO, absolute value of monocyte; NEU, absolute value of neutrophil; LYM%, lymphocyte percentage; NEU%, neutrophil percentage; NLR, ratio of neutrophil to lymphocyte; PT, prothrombin time; PTA, prothrombin activity; FIB, fibrinogen content; PCT, procalcitonin; CRP, c-reactive protein; ALT, alanine aminotransferase; AST, aspartate aminotransferase; TBIL, total bilirubin; ALB, albumin; Cr, creatinine; GFR, glomerular filtration rate; CO2CP, carbon dioxide combining power; CK, creatine kinase; CK-MB, creatine kinase isoenzyme-MB; CT, Computerized Tomography; GGO, ground-glass opacity; RR, respiratory rate; P/F, PaO2/FiO2; ICU, intensive care unit*.

a *p-values comparing severe and mild infection patients were calculated by Chi-square test and Fisher's exact test. Student's t-test was used where data were normally distributed (evaluated with Kolmogorov-Smirnov test), and non-parametric t-test (Mann-Whitney test) was used when data were not normally distributed. Statistical test differences were considered significant if the P-values were <0.05*.

### Clinical Indicators Associated With the Severity of COVID-19 Infection

Demographic and clinical data between mild and severe group were compared. Firstly, age was found strongly associated with the severity of diseases (45.34 ± 15.25 in mild vs. 64.75 ± 14.76 in severe group, *p* = 1.0E-06). Secondly, respiratory disease (*p* = 0.0067), cardiac disease (*p* = 0.0186), hypertension (*p* = 0.0011), and more than two comorbidities (*p* = 0.0024) were identified as the factors associated with the severity. Several biomarkers from the 1st laboratory detection were also identified as the potential factors related with the severity of the disease, including white blood cell count (4.15 ± 1.37 in mild vs. 6.08 ± 2.02 in severe group, *p* = 1.5E-04), absolute value of neutrophil (2.40 ± 1.25 in mild vs. 4.96 ± 2.41 in severe group, *p* = 3.3E-05), lymphocyte percentage (30.09 ± 11.51 in mild vs. 16.68 ± 9.41 in severe group, *p* = 3.0E-06), neutrophil percentage (59.03 ± 12.43 in mild vs. 74.67 ± 12.16 in severe group, *p* = 2.0E-06), ratio of neutrophil to lymphocyte (2.56 ± 1.99 in mild vs. 7.63 ± 8.32 in severe group, *p* = 6.9E-03), fibrinogen content (3.27 ± 1.03 in mild vs. 3.80 ± 0.98 in severe group, *p* = 0.041), c-reactive protein (20.30 ± 24.99 in mild vs. 58.59 ± 56.15 in severe group, *p* = 3.4E-03), total bilirubin (9.82 ± 4.19 in mild vs. 13.27 ± 6.89 in severe group, *p* = 7.4E-03), albumin (37.48 ± 3.88 in mild vs. 32.76 ± 5.37 in severe group, *p* = 4.5E-04), glomerular filtration rate (102.64 ± 24.16 in mild vs. 86.28 ± 24.06 in severe group, *p* = 6.8E-03), creatine kinase isoenzyme-MB (0.48 ± 0.65 in mild vs. 1.09 ± 1.17 in severe group, *p* = 2.3E-02), myoglobin (46.28 ± 33.53 in mild vs. 125.75 ± 123.48 in severe group, *p* = 4.8E-03), troponin (0.01 ± 0.01 in mild vs. 0.05 ± 0.08 in severe group, *p* = 3.0E-02). There was no significant difference in presenting symptoms and imaging of CT scan during the initial stage of COVID-19 infection between mild and severe groups. Demographic and clinical data are shown in Table 1.

### Scoring System for Prediction of Disease Severity in COVID-19

The factors associated with severity of COVID-19 in Table 1 were analyzed by univariate and multivariate logistic regression analysis. Age, pre-existing conditions (cardiac disease, hypertension, and more than two comorbidities), and 1st Laboratory detection (WBC, NEU, LYM%, NEU%, NLR, FIB, CRP, TBIL, ALB, GRF, CK-MB, Myoglobin, and Troponin) were identified as the predictors of the severity of disease by univariate analysis. Amongst them, age, WBC, NEU, GFR, and Myoglobin were selected by multivariate analysis as candidates of scoring system for prediction of disease severity in COVID-19 (Table 2). Each variable selected by multivariate analysis was assigned diverse scores according to their hazard ratio (HR). Patients with age above 59 years old were assigned a score of 1; and the level of WBC above 6.09, the value of neutrophil above 2.89 were given score of 2; GFR below 103.75 and myoglobin above 43 were assigned score 1. Finally, a scoring system was designed, which ranged from 0 to 7 by calculating each patient's score. Individuals with scores of 0–4 were defined to be at low risk of severity, and 5–7 at high risk (Table 3).

**Table 2:** Predictive factors for the severity of COVID-19 by Logistic Regression Model.

**Variables**	**Univariate**		**Multivariate**	
	**HR (95% CI)**	***P*-value**	**HR (95% CI)**	***P*-value**
Age	1.09 (1.04–1.13)	<0.001	1.08 (0.99–1.17)	**0.085**
Respiratory diseases	/	0.999		
Cardiac disease	5.89 (1.33–26.01)	0.019	0.21 (0.00–22.09)	0.514
Hypertension	5.92 (1.92–18.30)	0.002	0.35 (0.03–4.08)	0.399
More than 2 kinds	5.08 (1.69–15.22)	0.004	7.33 (0.37–146.07)	0.192
of diseases				
WBC	1.99 (1.41–2.81)	<0.001	1.88 (1.10–3.19)	**0.021**
Neutrophil	2.41 (1.59–3.64)	<0.001	1.72 (1.02–2.89)	**0.042**
LYM%	0.88 (0.82–0.94)	<0.001	1.07 (0.94–1.22)	0.285
NEU%	1.12 (1.06–1.18)	<0.001	1.16 (0.94–1.43)	0.161
NLR	1.55 (1.18–2.03)	0.001	1.17 (0.85–1.60)	0.336
Fib	1.64 (1.01–2.67)	0.045	0.52 (0.09–3.03)	0.470
CRP	1.03 (1.01–1.04)	0.001	1.01 (0.97–1.11)	0.574
TBIL	1.14 (1.02–1.26)	0.019	0.89 (0.71–1.13)	0.331
ALB	0.80 (0.70–0.90)	<0.001	0.87 (0.66–1.14)	0.322
GFR	0.97 (0.96–0.99)	0.013	1.05 (0.99–1.11)	**0.096**
CK-MB	2.30 (1.19–4.42)	0.013	1.22 (0.18–8.43)	0.844
Myoglobin	1.02 (1.01–1.04)	0.003	1.02 (0.99–1.04)	**0.094**
Troponin	/	0.001	/	0.670

*HR, hazard ratio; CI, confidence interval; WBC, white blood cell count; LYM%, lymphocyte percentage; NEU%, neutrophil percentage; NLR, ratio of neutrophil to lymphocyte; FIB, fibrinogen content; CRP, c-reactive protein; TBIL, total bilirubin; ALB, albumin; GFR, glomerular filtration rate; CK-MB, creatine kinase isoenzyme-MB*.

*The univariate and multivariate logistic regression analysis was employed to select variables potentially associated with severity of COVID-19 infection. Statistical test differences were considered significant if the P-values were <0.1. And the P-value was highlighted in bold if the difference is significant*.

**Table 3:** Scoring system for prediction of disease severity in COVID-19.

**Variables**	**HR**	**Score**
**Age**	1.08	
<59		0
≥59		1
**WBC**	1.88	
<6.09		0
≥6.09		2
**Neutrophil**	1.72	
<2.89		0
≥2.89		2
**GFR**	1.05	
<103.75		1
≥103.75		0
**Myoglobin**	1.02	
<43		0
≥43		1
Low risk		0–4
High risk		5–7

*HR, hazard ratio; WBC, white blood cell count; GFR, glomerular filtration rate*.

### Predictive Value and Validation of Scoring System to the Severity of COVID-19

**Figure 1:**
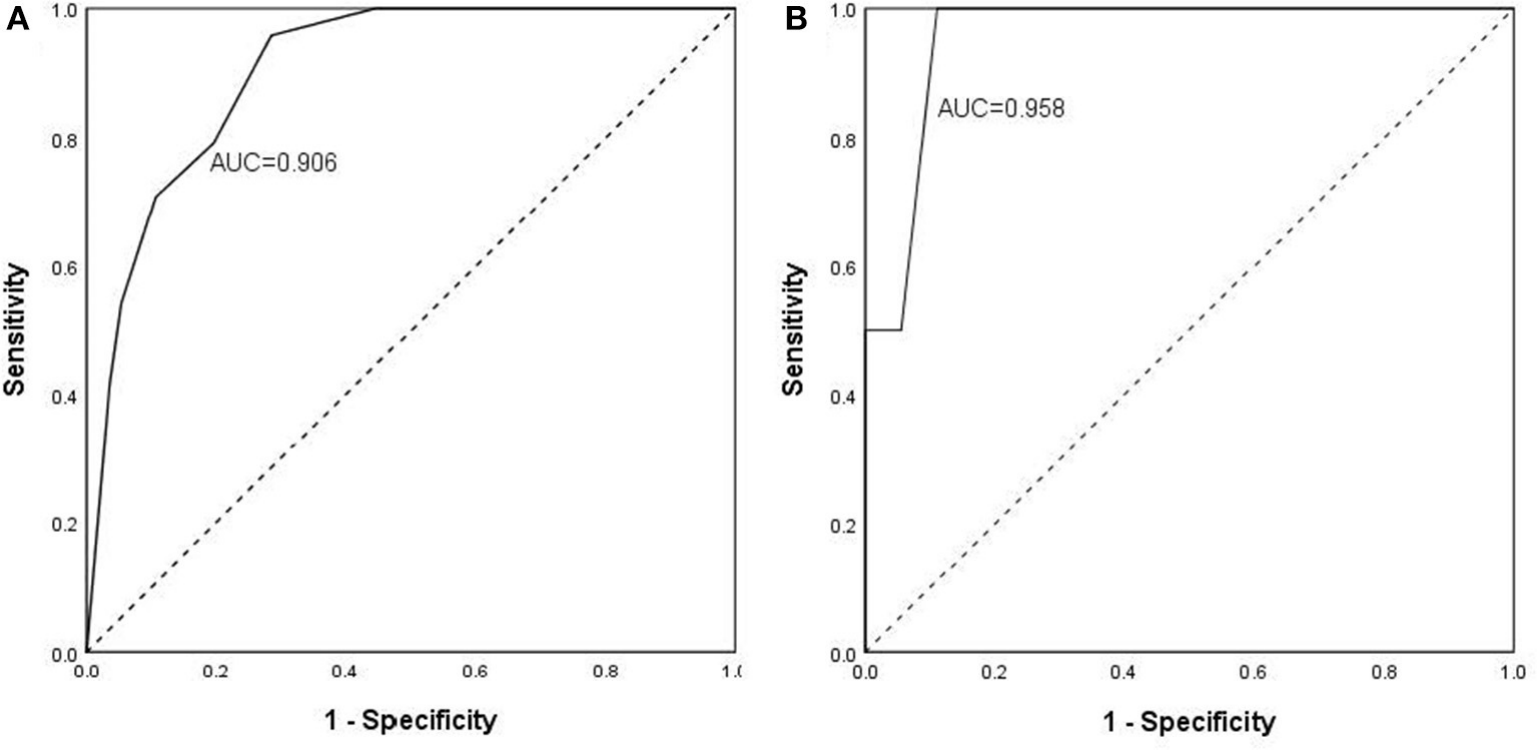
Predictive value and validation of scoring system to the severity of COVID-19. The scoring system was brought into the discovery cohort **(A)** to calculate the predictive value and found that the accuracy of prediction in severity. AUC is 0.906, sensitivity of prediction is 70.8%, and the specificity is 89.3%. The scoring system was brought into the validation cohort **(B)** to calculate the predictive value and found that the accuracy of prediction in severity. AUC is 0.958, sensitivity of prediction is 100%, and the specificity is 88.9%.

The scoring system was brought into the cohort to calculate the predictive value and found that the percentage of ICU admission (20%, 6/30) and ventilation (16.7%, 5/30) in patients with high risk was much higher than those (2%, 1/50; 2%, 1/50) in patients with low risk (*p* = 0.009; *p* = 0.026). The scoring system was then used to evaluate the accuracy of prediction in severity and found that the AUC is 0.906 (Figure 1A), sensitivity of prediction is 70.8%, and the specificity is 89.3%. The probability of patients in high risk group developing severe disease was 20.24 times than that in low risk group (*p* = 1.0E-06, Table 4). In addition, another 22 patients with COVID-19 were recruited from March to April of 2020 in the validation cohort. Amongst them, 18 patients were diagnosed as “mild” disease and 4 patients with “severe” disease. The variables from scoring system, including age, WBC, NEU, GFR, and Myoglobin were collected and the patients were divided into two groups (high risk vs. low risk) according to the scoring system. The accuracy of prediction in severity was evaluated and found that the AUC is 0.958, sensitivity of prediction is 100%, and the specificity is 88.9% (Figure 1B).

**Table 4:** Predictive value of scoring system to the severity of COVID-19.

**Variables**	**Low risk**	**High risk**	**OR**	***P*-value**
ICU	56/1	17/6	19.77	0.007
Ventilation	56/1	18/5	15.56	0.015
severity	50/7	6/17	20.24	1.0E-6

*OR, odds ratio; ICU, intensive care unit*.

*The prediction value of scoring system was determined by the area under the curve (AUC). Statistical test differences were considered significant if the P-values were <0.05*.

## Discussion

COVID 19 is a novel disease which has spread throughout the world and resulted in over seven thousand deaths worldwide in a few months. Most patients had mild symptoms with only 6.1% of patients progressing to severe disease requiring admission to ICU or the use of mechanical ventilation (Guan et al., 2020). There is an urgent need to find a simple and precise tool to predict the development of severity in COVID-19 infection at the early stage of disease (Wynants et al., 2020).

In the current study, we calculated a novel scoring system which could help predict the severity of COVID-19 infection from patient characteristics and clinical parameters collected on the first day of presentation to hospital. Although several factors, for example, age and NLR (Gong et al., 2020; Liu J. et al., 2020; Wang et al., 2020; Yang et al., 2020; Zhou et al., 2020) have previously been reported to be associated with the incidence of severe illness, we are the first to use scoring system to classify high and low risk of severity. We found that 63.33% of patients in the high-risk group developed severe infection, compared with only 10% of patients in low-risk group, which indicated that the hazard ratio of severity in high-risk group was 20 times of low-risk group. This will help set up different strategies for high and low risk group, which is very important for government to manage limited medical resources, also useful for patients to quell anxiety.

The second character of this scoring system is covering patients' condition, from pre-existing conditions to presenting symptoms. We found that pre-existing conditions, including respiratory disease, cardiac disease, hypertension, and more than comorbidities are risk factors strongly associated with the severity, although all of them were substituted by white blood cell count, absolute value of neutrophil, glomerular filtration rate and myoglobin in scoring system, which just indicates the importance of pre-existing conditions to the severity of COVID-19 infection. Amongst the five factors in scoring system, age is the basic factor of severity, which has become consensus in recent studies in COVID-19 (Gong et al., 2020) and Severe acute respiratory syndrome (SARS) (Chan et al., 2003) and Middle East respiratory syndrome (MERS) (Arabi et al., 2017). Moreover, several pre-existing conditions which are high-risk factors were reported by Gong et al. (2020), and in this study, we also found that these pre-existing conditions strongly associated with the severity for example, cardiac disease and hypertension, while they are rejected from the scoring system, because they are age-dependent factors.

In this study, white blood cell count and absolute value of neutrophil are selected to be the biomarker for predict the progress of the disease. The same as the other papers published previously, our data in the paper also found that the lymphocyte percentage descend with the disease, which indicates the direct result of viral infection (Dymond, 2018; Qin et al., 2019, 2020; Li et al., 2020; Liu Z. et al., 2020). And more interesting, we also found that the higher of white blood cell count and absolute value of neutrophil, the higher risk of severity, which give us a clue that abnormal virus-immune response cross talk in the early stage might affect the outcome of the disease (da Silva-Malta et al., 2017; Abd El-Kader and Al-Jiffri, 2018).

In addition, the biomarkers used in the scoring system are common and easily obtainable in an early stage of the disease (Havrilesky et al., 2008; Matthews et al., 2018). White blood cell count, absolute value of neutrophil, GFR and myoglobin are routine clinical detection in hospital, which could be get on the first day of hospital admission. The availability of these biomarkers indicates this scoring system could be used in an out-patient setting to classify patients in high or low risk of severity and receiving different therapy strategies.

In conclusion, our data clearly present a simple and precise scoring system to predict the possibility of severity in COVID-19 infection. Age, white blood cell count and pre-existing conditions could help calculate the score and further classify the risk of disease severity. Whilst the convenience of this scoring system is very important for current therapy during the period of pandemic of COVID-19 infection, further validation in large cohort is required.

## Data Availability Statement

The raw data supporting the conclusions of this article will be made available by the authors, without undue reservation.

## Ethics Statement

The studies involving human participants were reviewed and approved by Institutional Review Board of Beijing You'an Hospital. The patients/participants provided their written informed consent to participate in this study. Written informed consent was obtained from the individual(s) for the publication of any potentially identifiable images or data included in this article.

## Author Contributions

YZhang, RJ, and TD conceptualized and designed the study. KL, LQ, YZhang, YZhao, and QWanalyzed the data. CZ, LQ, KL, BX, LL, YD, YF, JS, XL, ZH, and HX helped with the clinical sample and data collection. YZhang and TD wrote, reviewed and revised the manuscript.

## Conflict of Interest

The authors declare that the research was conducted in the absence of any commercial or financial relationships that could be construed as a potential conflict of interest.
